# Proximate composition, mineral content and antinutritional factors of Brebra (*Millettia ferruginea)* seed flour as well as physicochemical characterization of its seed oil

**DOI:** 10.1186/2193-1801-3-298

**Published:** 2014-06-13

**Authors:** Berhanu Andualem, Amare Gessesse

**Affiliations:** Department of Biotechnology, Natural and Computational Sciences Faculty, University of Gondar, P.O. Box 196, Gondar, Ethiopia; Biotechnology Institute, Collage of Natural Sciences, Addis Ababa University, Addis Ababa, Ethiopia

**Keywords:** Amino acid, Antinutrional properties, Mineral content, Physicochemical characteristics, Proximate analysis

## Abstract

Still there is no scientific report about the proximate analysis of seeds and characteristics of oil produced from brebra seed. Objective of this study was to determine proximate and antinutritional characteristics of seeds as well as the physicochemical characteristics of brebra seed oil. Crude oil, protein, fiber, ash, moisture and carbohydrate content of brebra were 48.5 ± 0.99%, 29.7 ± 0.23%, 2.41 ± 0.12%, 3.24 ± 0%, 4.24 ± 0.04% and11.92 ± 0.2%, respectively. Seed has concentrated energy (6.0298 Kcal/gm). The respective tannin, oxalate and phytic acid value were 84.3 ± 0.89 mg/100 gm, 20.97 ± 0.36 mg/100 gm and 291.62 ± 0.87 mg/100 gm, respectively. Cyanide was not detected in the sample. Seed contains high concentration of phosphorus (1062.1 ± 0.3 mg/100 g), potassium (281 ± 0.1 mg/100 g), magnesium (112.38 ± 0.1 mg/g), sodium (93.26 ± 0.1 mg/g) and calcium (61.55 ± 0.01 mg/g). The oil was analyzed for specific gravity at 20°C, viscosity at 40°C, refractive index at 40°C, acid value, saponification value, iodine value, peroxide value and ester value. Their respective values were 0.942, 40.59 mm^2^/s, 1.473, 0.39 mg KOH/g, 174.95 mg KOH/g, 104.48 gI_2_/100 g, 6.88 and 174.56 mg KOH/g. Unsaturated fatty acids accounts (80.7%), of which 48.2% and 27.7% were linolcic and linolenic, respectively, which make suitable for production of biodiesel. Seed has higher nutrient composition, low antinutritional elements and high calorie value compared to some legumes.

## Background

*Millettia ferruginea* (Hoechst.) Baker is a useful endemic tree species of Ethiopia with great potential for agroforestry. It is belonging to the family Fabaceae (Leguminosae) sub-family Papilionnodeae. This plant is known to have two subspecies, namely, *ferruginae* and *darassana* (Thulin 1983). Subspecies *ferruginae* is known to occur at North Ethiopia within the range of 1,000 and 2,500 m above sea level, while subspecies *darassana* is located in the southern part of the country (particularly in Sidamo) within the range of 1,600 and 2,500 m above sea level. The hybrid of the two subspecies believed to be found in the central and western part of Ethiopia (Thulin 1983). According to the flora of Ethiopia, vol. 3:108, (1989), *Millettia ferruginea* found in the following regions*: subspecies ferruginea; Tigray, Gondar, Gojam, Shewa, Welega and Hareg. The* subspecies *darassana* is commonly found in Welega, Shewa, Harege, Bale, Ilubabor, Kefa and Sidamo. Any way both species are believed to be found only in Ethiopia, despite as yet an unconfirmed report from the Sudan (Thulin 1989).

*Millettia ferruginea* is a N_2_ fixing leguminous tree species that is known to have positive effects on associated crops in the southern parts of Ethiopia (Machachlan 2002). The tree usually occurs on farmlands in association with some important annual and perennial crops, such as barley (*Hordeum vulgare* L.), *Ensete ventricosum* Welw.) Cheeeman, maize (*Zea mays* L.), sorghum (*Sorgum bicolor* (L.) Moench s.l. and coffee (*Coffea arabica* L.) in the Wendo-Genet, Sugallae and Sokicha areas (Southern Ethiopia). According to Muleta (2007), from 14 dominant coffee shade tree species, *Millettia ferruginea* had the highest frequency of occurrence (22.3%). Soil near *Millettia ferruginea* tree is found to be rich in nutrient (Hailu et al. 2000). Its wood is used to fire wood, house construction, flowers serve as feed for bees; leaves, shoots and flowers are used as fodder for ruminants. The pulverized and crushed seeds widely used as fish poisons (Negash 2002) and as insecticides for scabies and chiggers (Stein 1973). It is also used for erosion control MacLachan (2002). Hailu et al. (2000), presented a reproduced summary of the use of *Millettia ferruginea* trees based on interviews made in Gedeo, Southern Ethiopia. Despite these significant benefits of the plant under this investigation, the plant seed protein and oil are not yet explored for production of economically important products like oil for soap and biodiesel production and oilcake for media and protein production for human and animal consumption. Currently, production of biodiesel from non-edible vegetable oil (like brebra oil) is considered important than that of using edible vegetable oil for biodiesel. Still there is no scientific report about the proximate analysis of seeds and characteristics of oil produced from brebra seed. Therefore, the main objective of this study was to determine proximate and antinutritional characteristics of seeds as well as the physicochemical characteristics of brebra seed oil. Such information may expand the scope of knowledge on the utilization and quality of the extracted oil and oilcake of the seed for different purposes.

## Results and discussion

### Sample preparation

In this study, the processes of harvesting of seeds and oil extraction methods were developed and optimized. Brebra is a name given to *Millettia ferruginea* in Amharic. This Amharic name was literally inherited from behavior of the mechanism of seed dispersal nature, which is the seed mechanically dispersed about 20 meters in average far from the tree in explosive manner. This nature of seed dispersal mechanism poses a problem for seed harvesting. To overcome harvesting problem, the matured pale yellow pods were collected from the tree and covered with *teff* straw for certain period of time to accomplish its maturity. After maturation, the pods were put into a fiber sac to facilitate aeration and dried there in the sac and lastly released seeds collected in the sac. Fiber sac can provide free ventilation of air in order to avoid deterioration of seed quality by fungi. This method was originally adopted from the society. It is well known that *Millettia ferruginea* contains a chemical compound that is found to be toxic for fish is known as rotenone (Dagne et al. 1990), which is widely used by the society for fishing. The society was collected the seeds and pods of the tree for fishing by the method already mentioned above.

**Figure 1:**
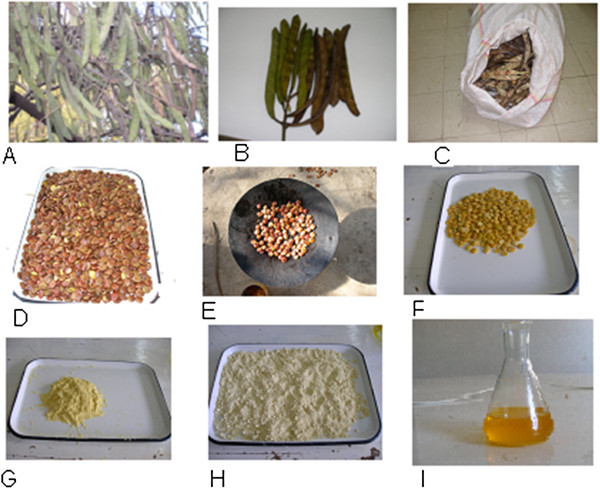
**The process of harvesting of seeds and oil extraction from brebra tree. A** = a typical brebra tree with pods, **B** = pod with seeds, **C** = pods in sac, **D** = harvested seeds, **E** = when seeds were dehulled by roasting with pan, **F** = dehulled seeds, **G** = powdered seeds prior to extraction of oil, **H** = defatted flour of brebra seed and **I** = extracted oil.

Out of 100 kg of dry pods with seeds, 25 kg (25%) seed was harvested. From an average sized tree, about 150 kg of pods containing seeds can be harvested. From a single tree it is possible to produce 37.5 kg seeds. From one hectare of land in average it is possible to plant about 35 trees. Therefore, from one hectare land one can harvest 1350 Kg dry weight of seeds. The whole process of harvesting and extraction of oil is shown on Figure 1. Pure oil was extracted by the help of co-solvent amended (hexane and ethanol) techniques. Ethanol was employed to remove any polar residues from oil. Ethanol soluble phospholipids, proteins and other polar substances in the oil were moved to the ethanol phase and pure oil remains in the hexane phase. Both solvents were recovered from their perspective mixture by means of Rota vapor.

### Proximate composition

Table 1 presents the result of the proximate chemical composition (% dry weight) of brebra seed. The results reveal brebra seed as an oilseed with a potential of high oil and protein content to satisfy calorie and protein demand of the populations. The extracted brebra oil with the average of 48.5% is in close agreement with the average value of 49.5% melon oil seeds (Lge et al. 1984), 48.1% pumpkin seed (Fagbemi & Oshodi, 1991), 48.9% conophornut (Enujiugha 2003), 49.1% cashew nut (Akinhanmi, et al. 2008), 50% castor seed, 50% sesame seed, but is higher than *Crambe abyssinica* oil seed, 45.4 (Massoura et al. 1996), 42% groundnut kernel, 37% rapeseed, 36% palm kernel, 35% mustard, 32% sunflower, 20% palm fruit, 13% cotton seed and 23.5% soybean (Paul and Southgate 1980). The very high oil content suggests that brebra can be used as potential source of raw material for commercial activities. In brief, it can serve as feedstock for production of biodiesel, glycerol, soap and economically important materials, but not as nutrition at this level unless further investigation is carried to remove mild toxic substance, rotenone.

**Table 1:** **Proximate chemical composition of non-defatted flour of brebra seed (gm/100 gm dry matter)**

Component	Mean ± S.D (%)	CV (%)
Crude oil	48.5 ± 0.99	2.04
Crude protein	29.7 ± 0.23	0.77
Crude fiber	2.41 ± 0.12	4.98
Ash	3.24 ± 0.0	0.0
Moisture	4.24 ± 0.04	0.94
Carbohydrate (by difference)*	14.32 ± 0.2	1.41
Dry matter	95.8 ± 0.07	0.07
Organic matter	92.52 ± 0.03	0.03
Nitrogen free extract (NFE)**	11.91 ± 0.2	1.68
Energy (Kcal/g)***	6.0298 ± 0.01	0.17
Rotenone	0.701 ± 0.02	2.85

All tests were performed in triplicates and mean values are taken.

*Carbohydrate = 100- (H2O + Ash + Cp + EE).

**NFE = 100 - (H_2_O + CP + CF + EE + Ash).

***Energy (kcal) = 4 (g protein + g carbohydrate) + 9 (g lipid).

CV% = S.D/mean × 100.

The amount of crude protein in brebra seed was 29.7%, which was higher than protein rich foods such as quinoa (Ogungbenle et al. 2009), bambara groundnut (Yagoub and Abdalla 2007), cowpeas (Ragab et al*.*^2004^), seeds ranging between 13.5-26.8%. Moreover, chick beans, 19.4%, lima bean, 19.8% (FAO, 1982), *Crambe abyssinica*, 25.1% (Massoura et al. 1996), pea, 20.1%, (Sumner et al. 1980) and cashew nut, 25.5% (Aremu et al. 2006), kidney beans, 20.9% and lentils, 22.9% (Perez-Hidalgo et al*.*^1997^) have less amount of proteins in comparison with that of brebra seed flour protein. On the other hand, the amount of brebra seed protein is almost equal to conophor nut, 29.1% (Enujiugha 2003), jack bean, 30.8% (Anonymous 1972), *Canaralia cathartica*, 31.2% (Seena and Sridhar 2006) and roselle (32.3%) (Mohammed et al. 2007) but less than lenti, 33.4% (Suliman et al*.*^2006^), *Cataralia maritime*, 34.1% (Seena and Sridhar 2006), ashew nut, 36.3% (Akinhanmi et al. 2008), soybeans, 37% (Messina 1997) and barbados, 48.1% (Yusuf et al. 2007). This high quantity of protein can serve as media for microorganisms, fed for animals and even can serve as human food after detailed investigation. The value obtained for carbohydrate (11.92%) in this study is incomparable with an acceptable range of values of legumes, 20-60% of dry weight (Arkroyed and Doughty 1964) but almost equal to carbohydrate content of conophor nut (Enujiugha 2003) and even greater than cashew nut (Akinhanmi et al. 2008). This result thus gave us indication that the energy source is largely oil and in some extent protein (through deamination).

The calculated metabolizable energy value (6.03/Kcal/g) is higher than 3.12 Kcal/gm in *C. palcherria* seed, 4.49 Kcal/gm in *G. affricanum* seeds (Ekop 2007) and 5.46 Kcal/gm in fish *Citharinun citharus* reported by (Abdullahi 1999) in Nigeria. The energy content of barbados (3.12 Kcal/gm) was smaller than brebra seed flour energy content Yusuf et al. (2007)). According to this finding, brebra seed has concentrated energy supply in comparison with the above reported energy sources.

The moisture value of the oil in this study which was 4.24% is somehow low when compared with the value of moisture of legumes ranging between 5.0% and 11% reported in the literatures (Aremu et al. 2006; Lge et al. 1984). Ash content of brebra seed, which is an indicator for mineral elements, in this study was 3.24%, which is closely comparable with ash values of 3.68%, 3.22% and 3.56% reported for pigeon pea, lima bean and lablab bean, respectively (Aletor and Aladetimi 1989). It has been recommended by Pomeranz and Clifto (1981) that ash contents of seeds and tubers should be in the range 1.5-3.5% in order to be suitable for animal feeds. In this case, the ash content of this study fall within this range hence it can be recommended for animal feeds and human consumption as well as it can serve as microbial media without mineral supplement.

The dry matter and organic matter content of brebra seed flour were 95.8% and 92.52%, respectively. These show that the seed flour contains high amount of organic matter. The amount of rotenone of the seed was 0.701 ± 0.02%. It can be used as insect pesticide if production and application method is developed.

Table 2 depicts the amino acid composition of brebra seed. Glutamic acid (18.62 g/100 g) was the most predominant amino acid followed by aspartic acid (6.43 g/100 g), leucine (2.98 g/100 g), and lysine (1.79 g/100 g). The values of amino acids showed that cysteine and methionine were in the lowest levels. On the other side, essential amino acids represented 11.88 g/100 g, while nonessential amino acids represented 32.12 g/100 g and E/N ratio was 0.37. Total amino acids of brebra (44.0 g/100 g) are greater than soybean total amino acids (36.62 g/100 g). However, total essential amino acids of soybean (13.52 g/100 g) are greater than brebra seed essential amino acids (11.88 g/100 g).

**Table 2:** **Total amino acid composition of brebra seed (g/100 g)**

Amino acids	Brebra seed	Soybean seed*
**Essential amino acids**		
Isoleucine	1.63	1.971
Leucine	2.98	3.309
Lysine	1.79	2.706
Methionine	0.12	0.547
Phenylalanine	1.75	2.122
Threonine	0.97	1.766
Histidine	0.70	1.097
**Non-essential amino acids**		
Alanine	1.33	1.915
Ornithine	0.02	-
Asparagine	1.60	-
Aspartate	6.43	5.112
Cysteine	0.09	-
Glutamate	18.62	7.874
Glycine	1.10	1.880
Proline	1.20	2.379
Serine	1.21	2.357
Tyrosine	0.52	1.539
Total free NEAA	0.04	-
Total free EAA	0.40	-
Total essential amino acids (E)	11.88	13.52
Total nonessential amino acids N)	32.12	23.10
E/N	0.37	0.59
Total AAS	44.00	36.62

*Source: USDA Nutrient database, 2010.

Amino acid profile of brebra seed protein was compared with the well-known amino acid composition of soybean. Total amino acid composition of brebra is far greater than the total amino acids of soybean (USDA Nutrient database 2010). It is rich in both essential and non-essential amino acids. As most edible legumes (Bhagya et al. 2007), lysine of brebra seed is higher than sulphur amino acids (cystine and methonine). Since brebra seed is rich in content of amino acids, it can use as a potential source of protein for human being.

### Mineral composition

Table 3 shows the mineral content of brebra seed. The abundant minerals were phosphorus (1062.1 ± 0.3 mg/100 g), potassium (281 ± 0.1 mg/100 g), magnesium (112.38 ± 0.1 mg/g), sodium (93.26 ± 0.1 mg/g) and calcium (61.55 ± 0.01 mg/g). All the mineral elements measured were found to be higher than conophor nut, cashew nut, and bean seeds (Table 3) (Akinhanmi et al. 2008; Aremu et al. 2006).

**Table 3:** **Mineral composition of defatted brebra seed flour**

Mineral	mg/100 g			
	Brebra seed	*Conophor nut	**Cashew nut Kerrel	***Ripened beans
Magnesium (Mg)	112.38 ± 0.1	57.37 ± 2.53	19.3 ± 0.1	28.7 ± 2.8
Calcium (Ca)	61.55 ± 0.01	42.06 ± 2.01	21.5 ± 0.0	140.0 ± 7.8
Sodium (Na)	93.26 ± 0.1	-	8.2 ± 0.2	60.8 ± 5.0
Zinc (Zn)	2.0 ± 0.2	6.84 ± 0.02	0.8 ± 0.1	10.7 ± 0.7
Iron (Fe)	27.81 ± 0	1.55 ± 0.08	0.6 ± 0.1	1.2 ± 0.1
Potassium (K)	281.00 ± 0.1	-	27.5 ± 0.4	1327.0 ± 2.3
Manganese (Mn)	25.5 ± 0.2	-	-	2.02 ± 0.1
Copper (Cu)	17.39 ± 0.1	1.56 ± 0.05	-	0.34 ± 0.1
Phosphorus (P)	1062.1 ± 0.3	465.95	14.0 ± 0.2	214 ± 14.1

*Enujiugha, 2003, **Akinhanmi et al. 2008, ***Bhagya et al. 2006.

In addition to its high protein content, brebra seed contains a high concentration of minerals, especially phosphorus, potassium, magnesium sodium and calcium. The mineral content of brebra seed is in general higher than those reported for other legumes (Akinhanmi et al. 2008; Aremu et al. 2006) as mentioned above. It has a potential to supply sufficient amount of minerals for consumers and microbial media for microorganisms.

### Antinutritional factors

Antinutritional components of brebra seed is summarized in Table 4. The presence of antinutritional factors in the sample is of significant importance since they are some deleterious effects on both humans and other animals, for instance, oxalate is a chelating agent, which binds calcium very effectively. Plants with high oxalate content may produce acute metabolic calcium deficiency (hypocalcemia) when we use plant product as a main food source (Checke and Shull, 1985). The concentration of oxalate (22.97 mg/100 gm) in the seed in this investigation seems to be on the low side when compared to reported values in some crop seeds (Umoren et al. 2005).

**Table 4:** **The antinutritional factors of brebra seed flour**

*Component*	Mean ± S.D (mg/100 gm)
Tannin	84.3 ± 0.89
Cyanide	B.D.L
Oxalate	20.97 ± 0.36
Phytic acid	291.62 ± 0.87

B.D.L = below detection limit.

Tannin is known to evoke growth-depressing effects in rats. In this study, the tannin level (84.3 mg/100 gm) was found to be relatively high in comparison with tannic acid found in some literatures (Akinyede et al. 2005; Enujiugha 2003; Umoren et al. 2005). However, the tannin content of this study is less than the tannin content of some dry bean seed varieties (930 mg/100 gm) (Deshpande et al. 1986). The tannin amount in this study may not be as such harmful as expected for consumption. High amount of tannins are well known to form complex with proteins and reduced the solubility of proteins and make protein less susceptible to proteolytic attack than the same proteins alone (Carbonaro et al. 1996). However, relatively some amount of tannin, like this finding, may have a potential role as protective factors against free radical mediated pathologies, such as cancer and atherosclerosis, in humans (Kehrer 1993). According to Bawadi et al*.* (2005) report, water-soluble condensed tannins extracted from black beans inhibited the growth of MCF-7, Caco-2 colon, and Hs578T breast as well as DU 145 prostatic cancer cells. Other findings, associating polyphenols (including tannins) to free radicals scavenging and metal chelating activities, suggested their potentially beneficial implications in the treatment and prevention of cancer (Hangen and Bennin 2002).

Cyanogenic glycoside contents of legume seeds have been investigated. According to Liener (1977) report, total cyanide values for different legumes such as cowpea, lima bean, field pea, kidney bean, chicken pea and pigeon pea, were 2.1, 210–312, 2.3, 2.0, 0.8 and 0.50 mg/100 gm, respectively. About 0.40 mg/100 gm of cyanogenic glycoside from *Milletia obanensis* was also reported by Umoren et al. (2005). In this study, the cyanide content was found to be below detectable level. In terms of cyanide content, brebra seed flour is safe to use as food for both humans and other animals. In summary, the antinutritional content of brebra seed flour is not out of the range value of different legumes seeds and other crops reported by other literatures.

### Physicochemical characterization of oil

The physicochemical properties of brebra are shown on Table 5. Oil is clearer brown yellow in colour and less viscous, 40.59 CTm at 40°C than cold break seed oil of 57.5 CTm at the same temperature (Catarelli et al. 1993). It has density and specific gravity of 0.942 and 0.926 at 20°C, respectively. The specific gravity of this study is lower than the specific gravity of cashew nut (0.964) (Aremu et al., 2006) and caster seed oil (0.958) (Akpan et al. 2007). The refractive index of this study (1.473) is in close agreement with 1.465 (Aremu et al. 2006), 1.462 (Akintayo and Bayer 2002) and 1.468 (Akpan et al. 2007) of castor, akee pulp and cashew nut seed oils, respectively. This implied that brebra oil is less thicker than most of drying oils whose refractive indices range from 1.475 to 1.485 (Duel and Tr 1951).

**Table 5:** **Brebra oil chemical and physical characteristics**

*Oil physicochemical characteristics*	Unit	Values
Colour		Pale-yellow color limpid liquid
Density at 20°C	Kg/m^3^	0.942
Specific gravity at 20°C	kg/l	0.926
Kinematic Viscosity at 40°C	mm^2^/s	40.59
Acid value	mg KOH/g	0.39
pH value		6.38
Saponification value	mg KOH/g	174.95
Refractive index at 40°C		1.473
Iodine value	gI_2_/100 gm	104.48
Peroxide value	mEq/Kg	6.88
Ester value		174.56

All values are mean value of triplicates.

The saponification value of the oil in this study was 174.95 mg KOH/gm. This was lower than the values for some common oils like castor seed oil (185.83) (Akpan et al. 2007), palm oil (196-205 mgKOH/g), groundnut oil (188-196 mgKOH/g), corn oil (187-196 mgKOH/g) as reported by Akinhanmi et al*.* (2008), coconut oil (253 mg KOH/gm) and palm kernel oil (247 mg KOH/gm) (Pearson, 1976). However, this saponification value is within the same range of some edible oils reported by Eromosele and Paschal (2002). Moreover, saponification value (174.95) of the oil in this investigation is almost within the range of (175–187) ASTM (2002) specification for oils. According to Pearson (1976), oils with lower saponification values contain high amount of long chain fatty acids. Therefore, the value obtained for brebra seed oil contained high quantity of higher fatty acids (as it is presented on Table 6, fatty acids ≥ 18 carbon chain accounts 92.8%). The value of viscosity of brebra oil was 40.49 mm^2^/s (as mentioned above) is found to be less than the viscosity of cashew nut oil (56 mm^2^/s) (Akinhanmi et al. 2008). For production of biodiesel, therefore, transesterification reaction is the best method to reduce the viscosity of the oil. The oil under investigation has very low acid value of 0.39 mg KOH/g when compared with cashew nut oil (0. 82 mg KOH/g) (Aremu et al. 2006), refined castor oil (0.869) and crude castor oil (1.148) (Akpan et al. 2007), plukenetia conophoora (11.5 mg KOH/g) as reported by Akintayo and Bayer (2002), aenniseed (47.6%) by Ohsodi (1992). The acid value in this oil is below the maximum limit (2.0 mg KOH/g) of DIN EN ISO 660 and nearly within the range of ASTM specification (0.4 - 4.0) of castor oil (ASTM 2002).

**Table 6:** Fatty acid composition of fatty acid of brebra oil (*FAs = fatty acids)

*No*	*Fatty acids*	*Carbon number*	*Quantity (%)*
	Unsaturated FAs*		
1	Oleic	C18:1	0.6
2	Linoleic	C18:2	48.2
3	Linolenic	C18:3	27.7
4	Arachidonic	C20:4	0.8
5	Eurcic	C22:1	3.4
	Total		80.7
	Saturated FAs*		
6	Palmitic	C16:0	7.2
7	Stearic	C18:0	1.9
8	Arachidic	C20:0	3.3
9	Behenic	C22:0	6.9
	Total		19.3

The low saponification value and acid value imply that the oil is more appropriate for biodiesel since the oil acid value for biodiesel, which is less than 1%. The iodine value of brebra oil was 104.48 gI_2_/100 gm (within the range, 100-120 gI_2_/100 gm, of DIN) which is greater than the range of 77–94 gI_2_/100 gm olive oil, 8–10 gI_2_/100 gm coconut oil, 12–18 gI_2_/100 gm palm kernel, 44–58 gI_2_/100 gm palm oil, 85–95 palme oleine, 20–45 gI_2_/100 gm palme stearine, 50–60 gI_2_/100 gm tallow, 60–70 gI_2_/100 gm lard, (http://dec2.tec.agrar.tu-muenchen.de/pflanzoel/rkstandarde.html) 44.4 gI_2_/100 gm cashew nut oil (Aremu et al. 2006), 38.1 gI_2_/100 gm citrullus vulgaris (Achinewhu 1990), *Hausa melon* seed oil (38.50 gI_2_/100 gm) (Oladimeji et al. 2001) and 84.8 gI_2_/100 gm refined castor oil (Akpan et al. 2007). One the other hand, the iodine value of this study was lower than the range of 110–115, 125–135, 125–140, and 115–124 gI_2_/100 gm value of rapeseed oil, sunflower oil, soybean oil and corn oil, respectively. The oil in this study is considered drying oil since drying oils have an iodine value above 100 gI_2_/100 gm (Duel and Tr 1951). The peroxide value of brebra oil was 6.88 mEq/Kg. It was greater than the peroxide value of 3.1 mEq/Kg of cashew nut oil (Aremu et al. 2006).

**Figure 2:**
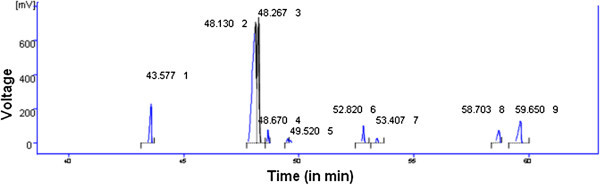
**Chromatogram results obtained from brebra oil.**

The fatty acid composition of brebra oil was determined by gas chromatography (Figure 2 and Table 6). The total unsaturated fatty acid content (80.7%) was higher than the total saturated fatty acids (19.3%) of brebra oil. Among polyunsaturated fatty acids linolcic (48.2%) and linolenic (27.7%) accounts 75.9% of the total fatty acids. The total unsaturated fatty acids (80.7%) of this study is greater than unsaturated fatty acids of 70.6% and 68.6% of cold break seed oil and hot break seed oil of tomato, respectively (Catarelli et al. 1993). Palmitic (7.2%) and behenic (6.9%) acids are the major saturated fatty acids in the oil under investigation. In summary, there is high degree of unsaturation with long chain fatty acids.

## Conclusion

From the result of the analysis, it can be shown that the seed flour of brebra has higher nutrient composition and calorie value compared to some legumes most especially in terms of crude oil and protein. The amount of protein in the seed is high in comparison with that of most protein rich crops. However, it may not serve as a good source for carbohydrates compared to other food sources. With regard to antinutritional studies, the antinutritional content of the sample under investigation is not out of the range value of different crops reported by other literatures. The percentage oil content of brebra seed was found to be 48.5%. The oil produced in this study was analyzed for specific gravity at 20°C, viscosity at 40°C, refractive index at 40°C, acid value, saponification value, iodine value, peroxide value and ester value. Their respective values are 0.942, 40.59 mm^2^/s, 1.473, 0.39 mg KOH/g, 174.95 mg KOH/g, 104.48 gI_2_/100 g, 6.88 and 174.56 mg KOH/g. The oil is also composed of 80. 7% unsaturated and 18.3%% saturated fatty acids. The physicochemical properties of the oil indicated that it is drying oil, which is rich in content of unsaturated fatty acids. Fatty acid composition of brebra seed oil make suitable for production of biodiesel and soap. These characteristics of oil make suitable for production of biodiesel and soap. Thus, oil is of good quality and could be recommended as suitable for industrial usage. To our knowledge, this is the first scientific report about the production and characterization of protein and oil from brebra tree, which is endemic in Ethiopia, by using standard oil test methods and standard parameters.

## Materials and methods

### Harvesting and sample collection

Harvesting process was adopted from traditional method of the society. Matured (pale yellow colored) pods of brebra from the study plant were collected and covered with the straw of teff (*Eragrotis teff*) for more than a week and then collected in the fiber sac, which is used to ventilate in order to avoid spoilage by fungi. The matured seeds were selected in order to improve the oil meal quality and to increase the capacity and efficiency of the extraction plant. The seeds were dried by using oven at 60°C more than 8 hr. The moisture content of the seed was determined by heating at 110°C for 24 hr in an oven by the procedure described by AOAC (1990). The seed coat of the seeds was dehulled by lightly roasted on pan and in the process water was added to sequester the seed coat and lastly dehulled by wooden mortar and pestle. For oil extraction, solvent (hexane) treatment techniques was used. To refine the oil co-solvent system technique (hexane and ethanol) was used. The process of refinery of the oil was determined and optimized in our previous study (Andualem and Gessesse 2012).

### Proximate analysis of seed

The methods used for sample treatment and analysis were carried out based on the standard procedures recommended by AOAC (1990). Crude fat, ash, total carbohydrates, total nitrogen and nitrogen free extract were determined according to AOAC (1990). Oil extraction was carried out by using hexane as a solvent. Brebra seeds were ground with blender (Waring blendor) and the fine flour was mixed with hexane and the whole content was stirred by magnetic stirrer for more than 4 hr and then filtered with Whatman’s No 1 filter paper. Hexane was recovered by the help of Rota vapor (Buchi, Switzerland) (Meher et al*.*^2006^) at 100 rpm. Total oil was quantified gravimetrically and calculated as percentage of oil. Protein (N × 6.25) was determined by the Kjeldahl method. To determine the ash content of the sample, 5 gm of the sample was incinerated in a muffle furnace. Crude fiber content of the sample was determined by mixing of the fine powder of the sample with 1.25% sulfuric acid and 1.25% sodium hydroxide solutions under specific conditions for ignition and dried residue remaining after digestion of the samples was considered as crude fiber (AOAC 1990). Calories were calculated by multiplying the amount of protein, carbohydrate and fat by the factors of 4, 4 and 9 (K cal) and 17, 17 and 37 (KJ), respectively, (EEC, 1990). To determine the moisture, the sample was dried to a constant weight in a vacuum oven at 100°C (AOAC, 1990). The moisture loss was determined gravimetrically.

### Determination of amino acid composition

#### Materials and reagents

The EZFaast GC-MS physiological amino acid analysis kit, Methanol (HPLC grade) and the internal standard and additional amino acid standards were obtained from Phenomenex (Cheshire, UK), (VWR, Leicestershire, UK) and Sigma (Dorset, UK), respectively.

#### Sample extraction (five replicates per treatment)

To determine total amino acid, 15 ± 0.03 mg sample was mixed to 1.00 ml 6 N HCl in 2 ml screw cap vial. The caps were taped in place with autoclave tape and heated in an oven at 110°C, 24 h. On removal from the oven, samples were cooled and 100 μL 0.75 mM norvaline solution added. Samples were mixed thoroughly and evaporated in a centrifugal vacuum concentrator (ThermoSavant). The residue was reconstituted in 1 ml distilled water and filtered through a 0.2 μM PTFE syringe filter (Pall Acrodisc) into a fresh vial. A 50 μl portion was transferred to a reaction tube (supplied with the EZFaast kit) and the solvent removed in a centrifugal sample concentrator (ThermoSavant).

In free amino acid analysis, 15 ± 0.03 mg sample was transferred to a 1.5 ml microfuge tube and 1 ml, 0.075 mM norvaline solution in 80:20 H_2_O: MeOH added. Samples were heated at 50°C, 10 min, and cooled for 2 min and centrifuged at 13400 rpm for 10 min. Lastly, 750 μL supernatant was transferred to a reaction tube (supplied with kit) and the solvent removed in a centrifugal sample concentrator (ThermoSavant). Sample residues were stored, desiccated at -18°C.

Samples were reconstituted in 0.2 ml 80:20 H_2_O: MeOH with vortexing to ensure complete dissolution. Amino acids were isolated from the samples by ion exchange and derivatized to their propyl-chloroformate derivatives according to the protocol supplied with the EZFaast’.

#### GC-MS analysis

Samples were analysed on a Hewlett Packard 5975C Inert MSD coupled to a 7890A Gas Chromatograph fitted with a Zebron Amino acid ZB-AAA column, split/splitless injector and MPS2 automatic liquid sampler. Two μl splitless injections were adjusted at purge time of 2 min with purge flow rate of 20 ml/min and an injector temperature was maintained at 220°C. The oven was programmed from 75°C (2.0 min) to 320°C (1.83 min) at 30°C/min was used with helium as the carrier gas at 9.5 kPa (1.4 ml/min), constant pressure. The source, quadrupole and transfer line temperatures were set at 230°C, 150°C and 320°C, respectively. Mass spectra were acquired at 70 eV over 45–450 m/z from 3–12 min with an acquisition rate of 3.5 Hz.

### Data analysis of amino acids

Data were quantified on the basis of extracted ion chromatograms (EIC) using the QuanLynx module of MassLynx 4.0 (Waters, Manchester, UK). The results were exported to Microsoft Excel (2003) and sample means and 95% confidence intervals (n = 5) were calculated for the free and total amino acid composition of the flour sample. Calibration curves from 0–26667 pmol.mg^-1^ F.W. and 0–2667 nmol.mg^-1^ F.W. (for the total and free amino acid respectively) were prepared and analysed alongside the samples.

### Mineral composition

To determine the mineral content of defatted flour, 5.0 g sample was incinerated in a furnace at 500°C and the residues dissolved in 50 ml of 2.5% HNO_3_ solution. The concentrations of Na, Ca, Mg, Fe, P, K, Zn, Cu, Fe, and Mn was determined using atomic spectrophotometer (Buck Science) absorption, following the method of Angelucci and Mantovani (1986). A calibration curve was prepared using standard metal solutions. Phosphorus was determined using the ammonium molybdate/ammonium vandate method of Chapman and Pratt (1968).

### Anti-nutritional factors of seed flour

#### Estimation of tannins

Tannins were estimated by Vanillin-HCl method of Price et al. (1978). Five gram of defatted brebra seed flour was treated with acidic methanol for extraction of tannins. From the diluted extract, 1 ml of was mixed with 5 ml of freshly prepared vanillin-HCl reagent and the optical density was determined at 500 nm by using spectrophotometer. As positive control, catechin standards were used side by side with the sample. The results were expressed as mg/100 gm dry wt.

#### Determination of phytic acid

Phytic acid composition was analyzed according to Wheeler and Ferrel (1971) by using 2.0 gm of dehydrated sample. A standard curve was constructed and expressed the results as Fe (NO_3_)3 equivalent. The amount of phytate phosphorus content was calculated from the standard curve by assuming that 4:6 iron to phosphorus molar ratio.

#### Determination of oxalate

To determine oxalate in brebra seed flour, the samples were separated into two fractions using the following procedure: two grams of finely grounded brebra seed flour was extracted with 100 ml of boiling distilled water for 30 min, filtered and adjusted to 200 ml. On the other hand, the hot water extract residue was further extracted with 150 ml of boiling 1 M HCl for 30 min, adjusted to 200 ml and filtered. The two filtrates were combined together. The content of oxalate in the two fractions was analyzed based on the method of AOAC (1990) with the help of potassium permanganate titration. All the analyses were tested triplicate and the results calculated and expressed on dry weight basis.

#### Determination of cyanide in brebra seed flour

The content of cyanide in brebra seed flour was determined by the amount of HCN released on hydrolysis. Brebra seed flour extract was obtained by homogenizing 30 gm of flour in 259 ml of 0.1 M orthophosphoric acid for 5 min. The homogenate was centrifuged at 2,500 rpm for 20 min and clear supernatant was taken. An aliquot of the supernatant was used for determination of hydrogen cyanide using an auto analyzer Technicon AAII, according to the method of Rao and Hahan (1984).

### Chemical characterization of brebra (*Meillettia ferrugeniea)*oil

After extraction of the oil, it was filtered to remove non-oil materials. A layer of sodium sulfate crystals was added to a flask and crude oil was added to remove any trace water. The dry agent was separated by decanting and filtration. The physicochemical determination of the oil for iodine value, saponification value and peroxide value were carried out according to the methods of AOAC (1990). Acid value was determined according to ASTM (2002). All tests were performed in triplicate.

#### Fatty acid analyses

The fatty acid profile was determined as fatty acid esters by gas chromatography. The sample methyl esters were prepared using the method used by the IOOC (International Olive Oil Council 2001). Standards of the methyl esters of the fatty acids were lauric (C12), myristic (C14), palmitic (C16), stearic (C18: 0), oleic (C18:1), linoleic (C18:2), linolenic (C18:3), arachidic (C20:0), arachidonic (C20:4), behenic (C22:0), eurcic (C22:1) and lignoceric (C24:0). Standard mixtures of these esters were injected in the Gas Chromatography (DANI GC 1000) for identification and quantification purposes. Standards and samples (3 drops dissolved in 3 ml of chloroform) were injected (0.5 ml) to the GC. The column and mobile phase were ECT-5 and 5% phenyl and 95% methylpolysiloxane, respectively. The flow rate and pressure used were ml per minute and 1.25 Bar, respectively. The GC oven was kept at 50°C for 2 min, heated at 4°C/min up to 250°C, where it was kept for 15 min. The detector was a flame ionization detector (FID), and the carrier gas was nitrogen (5 ml/min) (Alcantara et al. 2000).

The content of the sample was quantified by comparing the FID counts for each methyl ester of the GC sample of methyl ester with the FID counts of each methyl ester in the standard mixture of fatty acid methyl esters (FAME’s), averaging out these relationships for all the methyl esters (Alcantara et al. 2000).

#### Determination of ester value

The ester value is a measure of the amount of ester present in the given oil. It is expressed in the same terms as saponification value and the acid value. It was determined by subtracting the acid value from the saponification value (Ester value = Saponification value – Acid value).

### Physical characteristics of oil

Determination of physical characteristics such as moisture, specific gravity and density, Kinematic viscosity, refractive index and pH value were carried out according to the methods of ASTM (2002).

## Authors’ information

Bothe B-A and A-G are Associate Professor and most of the time we are engaged in research and management of different research projects.
